# Rewiring the Addicted Brain Through a Psychobiological Model of Physical Exercise

**DOI:** 10.3389/fpsyt.2019.00600

**Published:** 2019-08-27

**Authors:** Kell Grandjean Costa, Daniel Aranha Cabral, Rodrigo Hohl, Eduardo Bodnariuc Fontes

**Affiliations:** ^1^NEUROex: Research Group in Physical Activity, Cognition and Behavior, Center of Health Sciences, Federal University of Rio Grande do Norte, Natal, Brazil; ^2^Department of Physiology, Federal University of Juiz de Fora, Juiz de Fora, Brazil

**Keywords:** aerobic exercise, neuralplasticity, substance use disorder, addiction, alcohol abuse

## Abstract

Drug addiction is a worldwide public health problem, resulting from multiple phenomena, including those both social and biological. Chronic use of psychoactive substances has been shown to induce structural and functional changes in the brain that impair cognitive control and favor compulsive seeking behavior. Physical exercise has been proven to improve brain function and cognition in both healthy and clinical populations. While some studies have demonstrated the potential benefits of physical exercise in treating and preventing addictive behaviors, few studies have investigated its cognitive and neurobiological contributions to drug-addicted brains. Here, we review studies in humans using cognitive behavioral responses and neuroimaging techniques, which reveal that exercise can be an effective auxiliary treatment for drug addictive disorders. Moreover, we describe the neurobiological mechanisms by which exercise-induced neuroplasticity in the prefrontal cortex improves executive functions and may decrease compulsive behaviors in individuals prone to substance use disorders. Finally, we propose an integrative cognitive-psychobiological model of exercise for use in future research in drug addiction and practical guidance in clinical settings.

## Introduction

Addiction to psychoactive substances (e.g., nicotine, cocaine, marijuana, alcohol, heroin, inhalants, LSD, and ecstasy) is a public health problem of the modern world (1). The Diagnostic and Statistical Manual of Mental Disorders of the American Psychiatric Association (DSM-V 2013) classifies drug addiction as a substance use disorder (SUD) when an individual meets two or more of the following criteria regarding the use of psychoactive substances: tolerance, craving, repeated attempts to stop use, or social, personal, physical, or psychological problems related to drug use (2). In addition to the influences of biological, cultural, social, economic, and psychological factors on individuals with SUD (3), studies in animal models and humans have shown that psychoactive substance use induces epigenetic, molecular, structural, and functional changes to the brain (4). Thus, the neurobiological model of drug addiction has proposed a complex interaction between biological and environmental factors and created new integrative perspectives for prevention, treatment, and pharmacological targets (5).

SUD is traditionally related to abnormal dopamine release and sensitivity in the brain reward system. This neural network is composed of several interconnected brain areas, including the ventral tegmental area, nucleus accumbens, amygdala, striatum, hippocampus, and prefrontal cortex (PFC) (6). The PFC is an integrated neural system in humans required for normal executive functioning, including decision-making and inhibitory control, and beneficial socio-emotional functioning (7). Studies using positron emission tomography (PET) and functional magnetic resonance imaging (fMRI) have demonstrated that individuals with SUD present decreased activityin the PFC (8). This condition seems to be related to a reduced number of dopamine receptors and an abnormal firing rate of dopaminergic neurons (9). These changes in the dopamine system and PFC activity may favor compulsive substance intake and seeking behaviors, as well as loss of control over drug consumption (8). Similarly, incomplete prefrontal cortex development and the resulting decrease in ability to control impulsive decisions has been suggested as an explanation for adolescents’ particular vulnerability to drug abuse (10), highlighting the importance of preventing the use of addictive psychoactive drugs during this period of brain development. Hence, contemporary rehabilitation programs have emphasized the importance of interdisciplinary treatment approaches that target the reestablishment of normal PFC functioning while combining the use of medication, social care, and behavioral therapy supported by psychiatrists, psychologists, social workers, and family (5).

Physical exercise has been proposed as a complementary therapy for individuals with SUD undergoing treatment at different stages of addiction rehabilitation (11–13). Preclinical animal research has shown evidence of neurobiological mechanisms induced by physical exercise that support its potential use as a therapeutic strategy to treat drug addiction. Examples are the following: normalizing dopaminergic and glutaminergic transmissions, promoting epigenetic interactions mediated by BDNF (brain-derived neurotrophic factor), and modifying dopaminergic signaling in the basal ganglia (11, 14). However, identifying similar molecular interactions between exercise and the human brain presents significant methodological challenges that need to be overcome in order totranslate these findings from animal models to humans.

The benefits of physical exercise for cognitive functioning and brain structure in humans are, on the other hand, well documented in literature (15). For instance, aerobic exercise is linked to improvements in executive functions and increased gray matter volume and activity in PFC regions (16, 17). Furthermore, children and adults with higher cardiorespiratory fitness (i.e., VO_2_ max) show improved cognitive performance and neuronal activity in the PFC and anterior cingulate cortex (ACC) (18). The results of preclinical animal studies show that these brain adaptations seem to be related to the release of exercise-induced molecules, such as BDNF (19) and IGF-1 (insulin-like growth factor 1) (20). Both molecules act as neurotrophic factors and create new synapses, neurons, and neural networks (18). These adaptations are facilitated by an increase in cerebral blood flow during exercise (21) and a release of a vascular endothelial growth factor (VEGF) (22), which promotes mitotic activity in vascular endothelial cells, thereby promoting angiogenesis and enhancing the oxygen and nutrient supply to neurons (18). Additionally, exercise is also related to the integrity of the brain-blood barrier (23). However, despite the wide range of benefits of the exercising brain, its effects on individuals with SUD who have impaired PFCs and cognitive functions need to be further investigated.

In this mini review, we present the results of a review of the current literature on exercise and SUD. We limited our search to studies that investigated the effect of acute or chronic aerobic exercise on cognitive and/or neurobiological markers in humans with SUD. The search terms used to select the articles were “tobacco cigarettes,” “nicotine,” “alcohol,” “methamphetamine,” “crack,” “cocaine and marijuana,” “physical activity,” “endurance exercise,” “aerobic exercise,” “addiction,”:substance use disorder,” “executive functions,” “prefrontal cortex,” “cognition,” and “brain.” Two authors selected the published and peer-reviewed articles identified on electronic databases (Pubmed Central, Medline, Scopus, and Web of Science) in February 2019, while a third author resolved differences in opinion. Only articles published in English were considered. Finally, we propose an integrative cognitive-psychobiological model of exercise to support future research on the subject and provide methodological guidance for its application in clinical settings as a therapeutic tool for the treatment of SUD.

### The Effect of Aerobic Exercise on Brain and Cognitive Function in Individuals With SUD

Aerobic exercise is typically performed at submaximal intensity for a long duration with most of the energy consumption coming from mitochondrial oxygen-dependent production of ATP. Organic adaptations of the cardiorespiratory system as a result of aerobic training are mainly reflected by higher values of VO_2_ max, which has been associated with improvements in several health parameters, as well as brain and cognitive functioning (18, 24). Examples of aerobic exercise include running, swimming, and cycling among summer sports and cross-country skiing or speed skating among winter sports (25). **Table 1** describes studies that investigated the effect of aerobic exercise on the brain and cognitive functions in individuals with SUD. Acute effects of aerobic exercise (i.e., immediately after exercise cessation) have been shown to include increases in PFC oxygenation associated with greater inhibitory control (26) and improved memory, attention, and speed processing in polysubstance users (27). Similarly, methamphetamine users who exercised on a stationary cycling ergometer exhibited improvements afterward, such as better drug-specific inhibitory control, reduced craving levels, and enhanced brain activity in the ACC, the area involved in conflict monitoring and inhibition (28). Wang et al. (29) and Wang, Zhou, and Chang (30) also studied methamphetamine users and showed that exercise performed at moderate intensity (i.e., 65–75% of maximum heart rate) elicits a decrease in craving levels, improves performance on a go/no-go task, and increases N2 amplitude during no-go conditions when the individuals have to inhibit the impulse to press the bottom of the computer screen after a visual cue. Notably, the N2 is an event-related potential, monitored using non-invasive electroencephalography (EEG), that originates from the fronto-parietal cortex and is directly associated with inhibitory control (31).

**Table 1:** Studies investigating the effects of physical exercise on the brain and cognitive functions in individuals with substance use disorders.

Results from acute exercise studies					
Reference	Study procedures	Drug type	Exercise (type; intensity; time)	Neurobiological marker and cognitive test	Outcomes
Janse Van Rensburg and Taylor, (2008) (32)	Smokers (N=23) underwent to conditions (Exercise and passive resting). They performed a cognitive test before and after the conditions.	Nicotine	Aerobic exercise on a treadmill; Light self-paced intensity; 2min warm-up and 15min exercise	Stroop test	Following the exercise session, smokers did not improve on the cognitive test performance compared to the control session.
Janse Van Rensburg et al., (2009) (33)	Smokers (N=10) underwent to conditions (Exercise and passive resting) followed by fMRI scanning while watching smoking and neutral images.	Nicotine	Aerobic exercise on cycleergometer; Moderate-intensity (RPE 11-13); 2min warm-up, 10min exercise.	fMRI	Smokers presented reduced brain activity in areas related to reward, motivation and visuo-spatial attention following exercise, compared to the control condition.
Rensburg et al., (2012) ( 34)	Smokers (N=20) underwent to conditions (Exercise and passive resting) followed by fMRI scanning while watching smoking and neutral images.	Nicotine	Aerobic exercise on cycleergometer; Moderate-intensity (RPE 11-13); 2min warm-up, 10min exercise)	fMRI	Smokers presented decreased activity in visual processing (i.e., occipital cortex) areas during smoking images after the exercise session
Wang, Zhou and Chang., 2015 (30)	Participants (N=24) performed two conditions: exercise and reading control sessions The cognitive tests and the brain electroactivity were measured following each condition.	Methamphetamine	Aerobic exercise on cycle-ergometer; 65-75% of estimated maximum HR, 30min (5min warm-up, 20min of exercise and 5min cool-down)	Electroencephalogram (EEG), GoNoGo	Both general and methamphetamine specific inhibitory control were improved after the exercise session compared to the control session. Greater N2 amplitude was observed during the cognitive tests on the Nogo conditions of both inhibitory control tests compared to the control session.
Wang et al., 2016 (29)	Participants (N=92) were randomly assigned to 4 groups: light exercise, moderate exercise, vigorous exercise and reading control group. Cognitive test and brain electroactivity were measure before and 20min after the exercise or reading session.	Methamphetamine	Aerobic exercise on a cycle-ergometer; each group had its own intensity based on estimated maximum HR (40-50%, 65-75% and 85-95%, corresponding to light, moderate and high intensities, respectively); 30min of exercise (5min warm-up, 20min of exercise and 5min cool-down)	Electroencephalogram (EEG) a while performing a general GoNogo task and a methamphetamine specific GoNogo task.	Moderate intensity group showed better reaction time and lower number of errors. The same group showed greater N2 amplitude during Nogo conditions of both general and meth-specific inhibitory control.
Da Costa et al., 2017 (35)	Individuals with substance use disorder (N=15) were compared with 15 healthy individuals during a maximum effort exercise session. During the session, all volunteers had their prefrontal cortex oxygenation measured while performing a cognitive test.	Multiple drug users (35.5% were addicted to one substance, 43% to two substances and 21.1% to three substances). 8 reported to be crack/cocaine user, 6 were alcohol users and 3 were marijuana users.	Aerobic exercise until voluntary exhaustion [20 on Borg Scale (6-20)]. The cycloergometer was kept in 60-70 rpm. The initial load was 25w and in every two minutes, 25w increment occurred.	Near infrared spectroscopy (NIRS) and Stroop test	Individuals with substance use disorder increased prefrontal cortex oxygenation during exercise associated to better reaction time on the Stroop test. Also, lower cravings was reported after the exercise session.
Da Costa et al., (2016) (36)	Individuals with substance abuse (N=9) performed 3 months of exercise intervention. They performed a cognitive test before and after the exercise protocol.	Crack and cocaine	Aerobic exercise (free running), self-selected intensity; 3 sessions/week; 36-60min/session. The protocol lasted for 3 months.	Stroop test	It was found that the participants decreased the reaction time associated with improvements on cardiorespiratory fitness. The number of errors on the Stroop test kept the same comparing pre and post intervention.
Cabral et al., (2017) (37)(a)	Case report. The subject performed prefrontal cortex oxygenation during incremental exercise before, 45 days after and 90 days after the beginning of the running protocol.	Alcohol and nicotine	Aerobic exercise (free running); self-selected intensity; 3 sessions/week; the running time was increased along the weeks (first week: 3-6min, last week: 40-50min). The protocol lasted for 12 weeks.	Near infrared spectroscopy (NIRS). Stroop test	After 90 days of running, the subject improved prefrontal cortex oxygenation in 921% at ventilatory threshold, 604.2% at respiratory compensation point and 76.1% at maximum effort. Moreover, the individual increased number of correct answers during inhibitory control test by 266.6% and reaction time by 23%.
Wang et al., (2017) (38)	Randomized controlled trial study. Participants were divided in two groups: exercise (N=25) and control group (N=25). Cognitive tests and electroencephalogram were measured in both groups before and after 12 weeks.	Methamphetamine	Aerobic exercise (cycling, jogging, jump rope); 65-75% of estimated maximum HR; 3 sessions/week; 40min/session (5min warm-up, 30min of aerobic exercise and 5min cool-down). The protocol was conducted for 12 weeks.	Electroencephalogram (EEG), Go/NoGo	Both general and methamphetamine specific inhibitory control were improved after the exercise session compared to the control group. Greater N2 amplitude was observed during the cognitive tests on the Nogo conditions of both inhibitory tests compared to the control group.
Cabral et al., (2018)(39) (b)	Case report. The participant had its brain activity measured before and after the exercise protocol during rest, while doing a cognitive test. Moreover, prefrontal cortex oxygenation was measured during incremental treadmill exercise.	Crack/cocaine and alcohol	High intensity aerobic exercise; all out for 30s and resting for 4:30min 3 sessions a week. The protocol lasted for 4 weeks.	Electroencephalogram (EEG) and Near infrared spectroscopy (NIRS), Stroop test	Prefrontal cortex oxyhemoglobin increased 228.2% at the beginning of the treadmill test, 305.4% at the middle and 359.4% at the end of the test. Prefrontal cortex activity during the Stroop test was enhanced. The Stroop effect was decreased by 327%.

In nicotine users, a meta-analysis (40) and a systematic review (41) show little or no effect of exercise in smoking cessation. However, those reviews did not include studies using cognitive or neurobiological markers as outcomes. On the other hand, Rensburg et al. (32–34) conducted a series of important experiments that suggest potential benefits of aerobic exercise to the brain and cognitive functions of nicotine users. The first study showed that 15 min of light-intensity treadmill exercise reduced craving levels compared to a control condition (passive resting) but did not find improvements in inhibitory control. However, performance on the inhibitory control task was only measured by reaction time and not by the number of errors, which might limit our interpretation of the results (32). In the second experiment, 10 min of moderate-intensity cycling exercise elicited decreases in craving levels compared to a control condition (passive sitting for 10 min). After each condition, participants underwent fMRI scanning while viewing neutral pictures and pictures related to smoking. While viewing smoking images participants demonstrated reduced activation in brain areas related to reward (i.e., caudate nucleus), motivation (i.e., orbitofrontal cortex), and visuo-spatial attention (i.e., parietal lobe and parahippocampal gyrus) after exercise (33). Another study replicated the same experimental design with a larger sample of smokers. The results showed that 10 min of moderate-intensity exercise also reduced craving levels, and the fMRI analyses revealed decreased activity in visual processing (i.e., occipital cortex) areas during smoking images for the exercise condition but not for the control condition (passive sitting) (34). Thus, these results show the potential effects of aerobic exercise in modulating craving and correlated brain areas in nicotine users.

Therefore, despite the limited amount of studies available in the literature so far, it is apparent that acute sessions of aerobic exercise decrease craving levels and seem to benefit cognitive and brain functions in these individuals. However, it could also be important to understand if regularly performed exercise (i.e., chronic effects) may potentialize the acute benefits to the brain and cognition of individuals with SUD throughout weeks and months of exercise training. To date, only two studies have investigated the chronic effects of aerobic exercise in individuals with SUD using neurobiological and cognitive markers (**Table 1**). In one study, methamphetamine users showed improved inhibitory control and greater activation of the ACC during an inhibition task after performing 3 months of moderate-intensity exercise for 30 min three times a week (38). Curiously, this pioneering work by Wang et al. (38) did not report changes in cardiorespiratory fitness, which limited the association between the cardiorespiratory adaptations induced by exercise and improvements in brain and cognitive functioning. However, the results of a different pilot longitudinal study with polysubstance users showed that 3 months of aerobic exercise improved inhibitory control and was correlated with cardiorespiratory fitness improvements (36).

Because of the lack of longitudinal studies in the literature, we have conducted two case reports, in which we tested two different exercise interventions. The first one was a 3-month running program (three times a week), based on self-selected moderate-intensity exercise. The study was conducted with a chronic alcohol user receiving treatment in a public psychiatric hospital. Measures of PFC oxygenation, inhibitory control, and the need for medical intervention were assessed before and after the exercise program. At the end of the 3-month period, the participant demonstrated improved PFC oxygenation, decreased reaction time in the inhibitory control task, and reduced need for medical intervention (37). The second case report involved a crack/cocaine and alcohol user receiving treatment. They engaged in 4 weeks of high-intensity exercise (three times a week), and we measured PFC oxygenation, brain activity through electroencephalography, and inhibitory control before and after the intervention. The participant showed increased PFC activity during the inhibitory control test and increased PFC oxygenation during exercise (39). Taken together, the relationship between cognitive abilities and brain function and regular exercise suggests a promising role of physical exercise in promoting greater executive control on the compulsive behavior of individuals with SUD.

## Psychobiology of Self-Selected Exercise Intensity: Practical Tool for Clinical Settings And Research

From an evolutionary perspective, humans have adapted to withstanding prolonged aerobic exercise through the search for food and persistence hunting of prey (supposedly pursued until physical exhaustion) (42). Aerobic self-selected exercise along with the cognitive appraisal of environmental cues for the acquisition of food and survival have been postulated to be key features in the development of the human brain (43). However, modern society has removed the need for humans to run/walk for food or shelter. As a result there is an increasing rate of hypokinetic behavior and related diseases such as diabetes, obesity, and hypertension (44, 45). Rational declarative decision-making concerning the volume, intensity, and frequency of exercise has not been sufficient to change sedentary behavior. Therefore, methods are being proposed to promote greater adherence to physical activity regiments, and a psychobiological integrative perspective appears to be a promising approach to achieve this goal (46, 47).

Cognitive and affective regulation of exercise intensity have been suggested to play a key role in both tolerance and adherence to exercise programs. For instance, homeostatic disturbances caused by high-intensity exercise have been associated with negative affective states and lower pleasure during exercise in sedentary individuals (45), leading to lower rates of adherence (48). Conversely, self-selected exercise intensity has been associated with positive affective states and higher levels of pleasure during exercise (45). Self-selected exercise intensity emphasizes the brain as the central governor of exercise intensity fluctuations (46), whereas the decision-making to increase and decrease velocity or tolerate or terminate the exercise session is controlled by the PFC through a bi-directional mind/body integration (49). Within this framework, top-down mechanisms are those initiated *via* declarative or non-declarative mental processing at the PFC level, which regulates muscle recruitment and alters physiological and behavioral responses. On the other hand, bottom-up mechanisms are initiated by sensitizing the ubiquitous somato-, viscero-, chemo-, and mechanical sensory receptors that influence central neural processing from the periphery to the brainstem, limbic system, and cerebral cortex (50). While performing any physical activity with self-selected intensity, the cognitive interpretation of the physiological state may be constantly working to preserve body homeostasis in order to reach the established goal (46, 51). In other words, fluctuations in pace while running are a behavioral outcome monitored by the brain (52). This behavioral modification results from integrating the task cognitive appraisal with afferent information related to biochemical and biophysical changes, such as temperature, heart and respiratory rate, blood pressure, blood concentrations of metabolites (e.g., PO_2_, PCO_2_, H^+^, HCO_3_^−^, and lactate), intramuscular H^+^, and energy substrate availability during the exercise (53).

**Figure 1:**
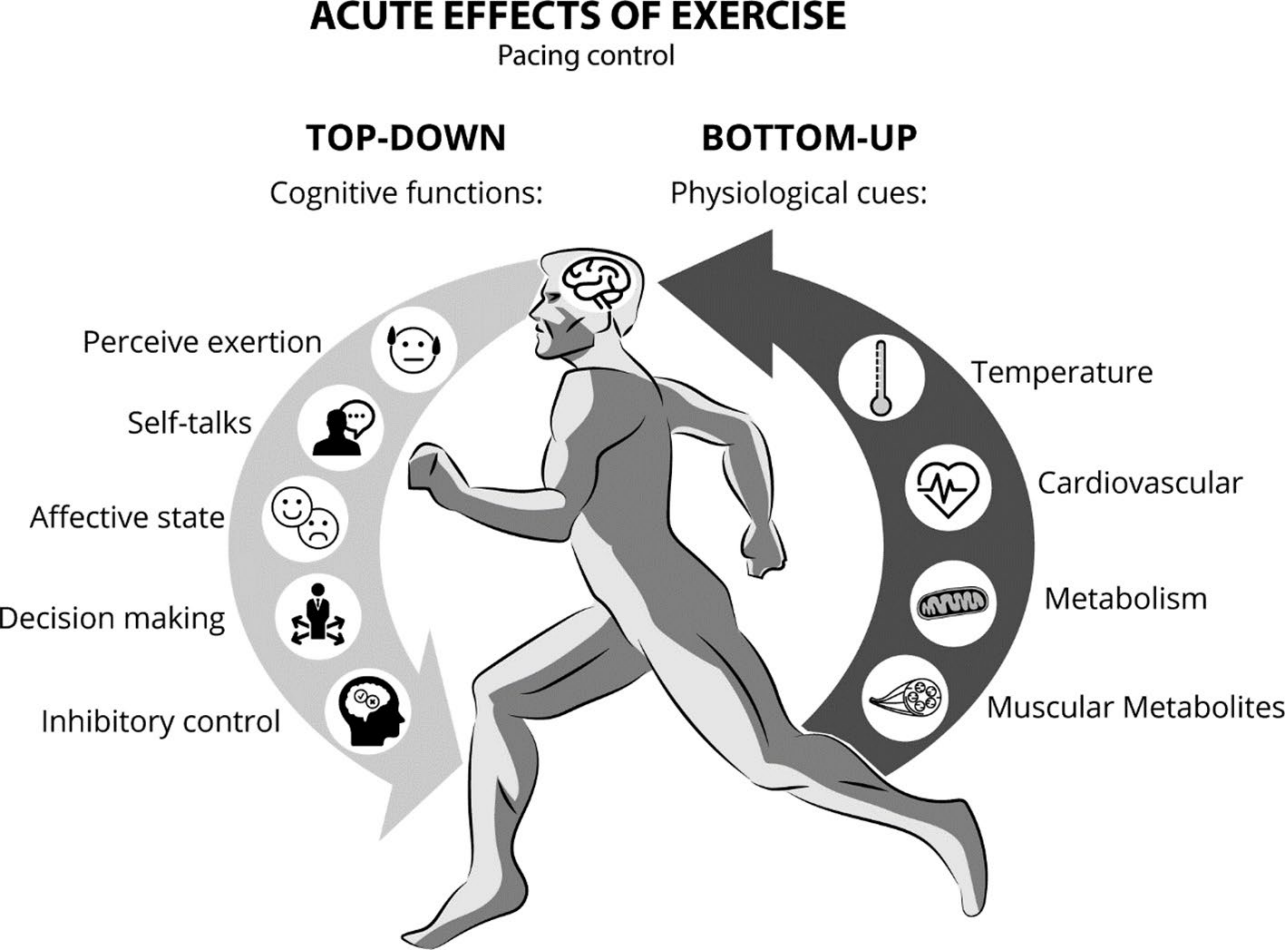
Pace control during continuous exercise while integrating top-down (cognitive functions) and bottom-up processing factors (physiological responses).

Furthermore, feelings of fatigue and self-defeating thoughts demand inhibitory control mediated by the PFC in order to maintain physical activity (54). In this context, decision-making might be based on feelings such as perceived exertion (i.e., how hard the exercise is), affect (i.e., generic valence for good and bad feelings), and internal conversations such as “I cannot do it,” “I will give up,” or “it is very difficult” (53, 55). Therefore, self-selected exercise intensity emphasizes cognitive control (top-down) under the physiological changes (bottom-up) during physical effort (**Figure 1**), and it can be used as a strategy to develop self-monitoring and self-control abilities during the treatment of individuals with SUD. For instance, when setting a goal during an exercise session, such as running for a specific time or distance (i.e., time trial exercise), individuals need to regulate their pace to successfully complete that task. Thus, during the exercise, the decision to regulate the pace (running velocity) will be influenced by several environmental stimuli (i.e. weather, terrain, competitors, verbal instructions, and time or distance feedbacks) combined with the physiological state.

Several therapies focusing on this mind-body interaction through the top-down and bottom-up bi-directional mechanism have been suggested as promising rehabilitation tools in regulating stress and the immune system (56, 57). Therefore, we hypothesize that self-selected exercise intensity employs the bi-directional mechanism enabling improvements in self-control abilities associated with brain exercise-induced neuroplasticity. This cognitive regulation can be tested in humans while investigating perceptual responses, exercise-induced effects, and PFC function using neuroimaging methods (e.g., fMRI, PET scan, and fNIRS) and/or electroencephalogram. In addition, the brain responses can be associated with tests that evaluate the executive constructs of SUD-specific decision-making and inhibitory control, such as cue-reactivity go/no-go tests in which individuals have to inhibit their responses to salient stimuli relating to drug-related cues (e.g., drug behavior pictures). This cue-reactivity response has been shown to activate areas of the PFCand to predict relapses in different substances disorders (58, 59). Thus, we suggest that randomized clinical trials could follow the neuroscience paradigm and cognitive methodologies to test this hypothesis. In addition, the implementation of a control group would play a key role in these experimental designs in order to compare the self-selected intensity of exercise with other types of exercise intensity regulation to demonstrate its efficacy.

## Conclusion

Despite the need for further prospective studies and clinical trials to test the efficacy of the psychobiological model of exercise as an intervention and treatment for SUD, physical exercise has been shown to be an effective and promising additional therapeutic tool for individuals with SUD. Here, we have described the brain areas affected by chronic substance use in patients with SUD as well as those improved by aerobic exercise. Some of these areas are primarily related to executive functions, which refer to a set of self-regulatory processes associated with the control of thoughts and behavior, including inhibitory control and decision-making. Therefore, in the same way that physical exercise is advised for treating other diseases, the neuroplasticity promoted by aerobic exercise may indicate its usefulness as a potential additional treatment for individuals with SUD. Specifically, these benefits may be seen in brain areas related to executive control, such as those areas involved in inhibition of drug-seeking behavior and impulsivity, as well as in decision-making regarding drug consumption. Furthermore, individuals with SUD who improve their fitness levels may enhance PFC function and cognition. These benefits should improve an individual’s ability to inhibit drug consumption behavior when exposed to environmental cues and, consequently, their ability to maintain abstinence. However, this is still a hypothesis, and further studies are necessary to provide evidence of the effectiveness of exercise on maintaining drug abstinence, specifically exercise of self-regulated intensity. Thus, we propose an integrative cognitive-psychobiological model of exercise for future research and provide practical guidance to optimize its potential benefits during rehabilitation programs.

## Author Contributions

KC and EF conceived the idea, draft, figure and final revision. DC reviewed literature for table, described the results and final revision. RH reviewed manuscript and added theoretical framework, practical application and final revision.

## Conflict of Interest Statement

The authors declare that the research was conducted in the absence of any commercial or financial relationships that could be construed as a potential conflict of interest.
